# Comparing Models for Early Warning Systems of Neglected Tropical Diseases

**DOI:** 10.1371/journal.pntd.0000033

**Published:** 2007-10-22

**Authors:** Luis Fernando Chaves, Mercedes Pascual

**Affiliations:** Department of Ecology and Evolutionary Biology, University of Michigan, Ann Arbor, Michigan, United States of America; Swiss Tropical Institute, Switzerland

## Abstract

**Background:**

Early warning systems (EWS) are management tools to predict the occurrence of epidemics of infectious diseases. While climate-based EWS have been developed for malaria, no standard protocol to evaluate and compare EWS has been proposed. Additionally, there are several neglected tropical diseases whose transmission is sensitive to environmental conditions, for which no EWS have been proposed, though they represent a large burden for the affected populations.

**Methodology/Principal Findings:**

In the present paper, an overview of the available linear and non-linear tools to predict seasonal time series of diseases is presented. Also, a general methodology to compare and evaluate models for prediction is presented and illustrated using American cutaneous leishmaniasis, a neglected tropical disease, as an example. The comparison of the different models using the predictive *R* ^2^ for forecasts of “out-of-fit” data (data that has not been used to fit the models) shows that for the several linear and non-linear models tested, the best results were obtained for seasonal autoregressive (SAR) models that incorporate climatic covariates. An additional bootstrapping experiment shows that the relationship of the disease time series with the climatic covariates is strong and consistent for the SAR modeling approach. While the autoregressive part of the model is not significant, the exogenous forcing due to climate is always statistically significant. Prediction accuracy can vary from 50% to over 80% for disease burden at time scales of one year or shorter.

**Conclusions/Significance:**

This study illustrates a protocol for the development of EWS that includes three main steps: (i) the fitting of different models using several methodologies, (ii) the comparison of models based on the predictability of “out-of-fit” data, and (iii) the assessment of the robustness of the relationship between the disease and the variables in the model selected as best with an objective criterion.

## Author Summary

Early Warning Systems (EWS) are management tools to predict the occurrence of epidemics. They are based on the dependence of a given infectious disease on environmental variables. Although several neglected tropical diseases are sensitive to the effect of climate, our ability to predict their dynamics has been barely studied. In this paper, we use several models to determine if the relationship between cases and climatic variability is robust—that is, not simply an artifact of model choice. We propose that EWS should be based on results from several models that are to be compared in terms of their ability to predict future number of cases. We use a specific metric for this comparison known as the predictive *R* ^2^, which measures the accuracy of the predictions. For example, an *R* ^2^ of 1 indicates perfect accuracy for predictions that perfectly match observed cases. For cutaneous leishmaniasis, *R* ^2 ^values range from 72% to77%, well above predictions using mean seasonal values (64%). We emphasize that predictability should be evaluated with observations that have not been used to fit the model. Finally, we argue that EWS should incorporate climatic variables that are known to have a consistent relationship with the number of observed cases.

## Introduction

One of the best documented patterns in the dynamics of vector-transmitted diseases is their periodicity at seasonal and interannual temporal scales [1–7]. These periodicities are the basis for the proposal that early warning systems (EWS) are feasible and useful tools for planning and decision making [2]. EWS are alert systems whose objective is to predict either epidemic outbreaks in regions where disease transmission is unstable or large outbreaks where the disease is endemic. From the early 1910s, when Captain S. R. Christophers of the British army developed a system to predict malaria in India using climatic and socioeconomic data [8,9], to present times when systems are based on indoor resting densities of vectors [10], climate, land use, and satellite imagery [11], EWS have been regarded as useful tools to help the development of poor and disease-stricken nations [2,11]. The early experience by Christophers was highly successful, and his system was in use until the 1940s, when the importance of malaria as a public health issue in the Indian subcontinent diminished [9,11]. However, recent results have demonstrated that the blind use of EWS can lead to unreliable forecasts, especially when models are used in regions where the connection between climate and disease is not well understood [12].

Despite the possible caveats of climate-based EWS, especially because of the complexity of human diseases for which social components can be as important as natural forces [13–15], there are successful examples of prediction of “out-of-fit” data based on the known association between climate and disease [6]. Although most of the effort in developing EWS has been focused on malaria [1,2],[16], similar efforts would be valuable for neglected tropical diseases, which represent a large burden for developing countries and whose transmission is sensitive to climate variability [6,17]. The leishmaniases in particular represent the fourth most important neglected tropical disease, with a burden of at least 2.1 million infected people per year, second to malaria in terms of the number of people affected by a protozoan vector-transmitted disease [17,18]. Like many other diseases, the infections are caused by protozoa, belonging to any of several different species of *Leishmania* spp. (Kinetoplastida: Trypanosomatidae), transmitted by sand flies (Diptera: Psychodidae). The clinical manifestation encompasses visceral and cutaneous/mucocutaneous cases, and is associated with a certain parasite species [6]. Our previous results indicate that American cutaneous leishmaniasis (ACL) is a good candidate for the use of climate-based EWS, because predictions with seasonal autoregressive (SAR) models can have an accuracy of over 75% [6]. Our objective in this paper is to illustrate a protocol for the development of EWS, including the evaluation of different linear and non-linear techniques for time series modeling and prediction, as well as assessment of the robustness of the relationship between the disease and climate that is the basis for building EWS.

## Methods

### Data

#### Leishmaniasis

Monthly records of ACL cases from January 1991 to December 2001 were obtained from the epidemic surveillance service Vigilancia de la Salud, of Costa Rica. The data were normalized using a square root transformation.

#### Climatic Covariates

**Figure 1:**
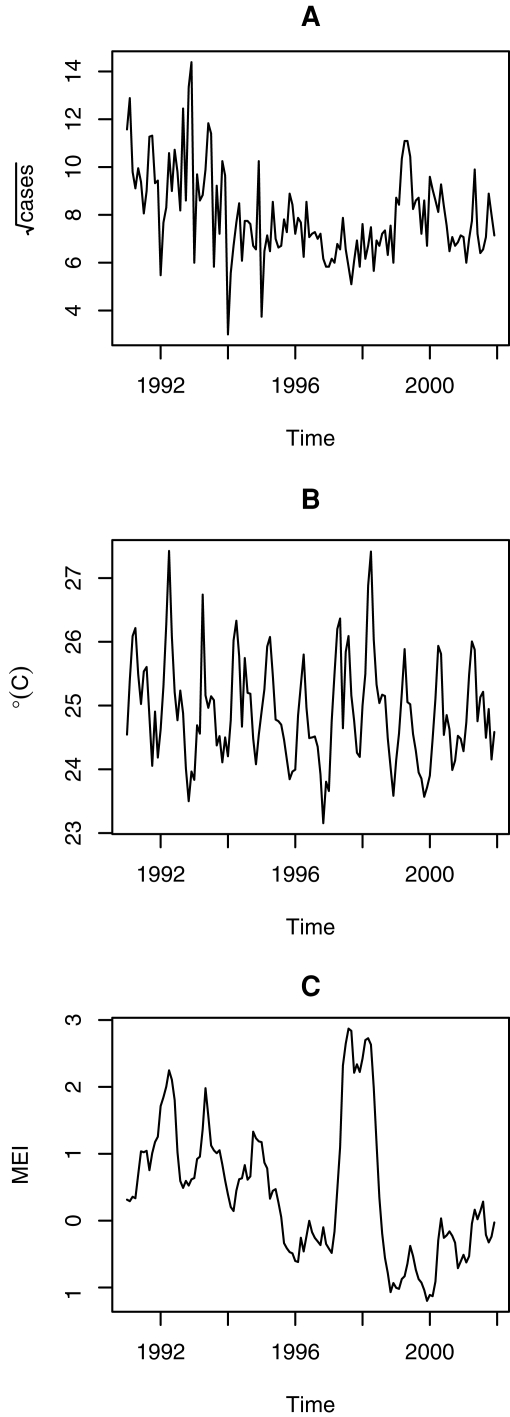
Time Series. (A) Square root Transformed ACL Cases in Costa Rica. (B) Mean Temperature in Costa Rica. (C) MEI.

The temperature (*T*) data are those used in [6] consisting of the average temperature in the 0.5°×0.5° grids composing the Costa Rica land surface [http://www.cru.uea.ac.uk,19]. The monthly average of these temperature records, *T*, and the multivariate ENSO index, *MEI*, [http://www.cdc.noaa.gov/people/klaus.wolter/MEI, 20] were used as predictors for modeling the transformed ACL cases. For all the models below, except for the non-linear forecasting (NLF) and the basic structural model (BSM), the lags for the introduction of climate covariates *T* and *MEI* were chosen based on our previous results using cross-correlation functions [6], with a fixed delay (i.e., months preceding the cases series) of 13 months for *MEI* and 4, 8, and 20 months for *T*. All time series are shown in Figure 1. Other climatic covariates, precipitation and relative humidity, were ignored since they did not show a strong association with the case data using non-stationary tools like wavelet cross-coherence [6].

### Statistical Analyses

#### Forecasting models

Several linear and non-linear models were fitted to the square root transformed case data. Brief descriptions follow of: (1) the approach to handle seasonality, (2) the types of models used, and (3) their classification as linear or non-linear.

#### Seasonality

To introduce seasonality, the strategy for all models was to include lags 12 and 13 of the transformed case data. This approach was chosen because the autoregressive treatment of seasonality is known to be the best approximation to the asymptotic cyclical structure of a time series [21]. This approach specifically allows a better minimization of the error variance when compared to a fixed seasonality implemented with a standard cyclical function (such as sines or cosines) that leads to a symmetrical cyclical structure [21].

#### Linear

In this class of models, parameters have a linear relationship with the response variable 22], in this case the transformed number of cases. This definition should not be confused with that of a linear dynamical system where the relationship of the dependent variables or covariates is linear with that of the independent variable [23]. In fact, linear models can be used to fit the parameters of non-linear dynamical systems, provided that the relationship between a response (which can be a transformation of the independent variable in the non-linear dynamical system) and the covariates (which also can be transformed) is linked by a parameter linearly. Linear models used in this paper include SAR and BSM.

#### Non-Linear

In these models, the relationship between the response and the parameters for the predictors is not constrained to be linear. Models include NLF, generalized additive models (GAM), and feed-forward neural networks (FNN). A description of the methods (linear and non-linear) and of the fitted models can be found in Protocol S1].

#### Forecasts

For all models, forecasts were obtained for prediction time intervals of 1, 3, 6, and 12 months ahead for a total of 24 months each. Each model was refitted recurrently before computing the next prediction by including all the previous months in the series [6]. The accuracy of the forecast was measured using the predictive *R* ^2^, which has an interpretation similar to the *R* ^2^ of a linear regression by definition [23] and is obtained as *R* ^2^ = 1–(mean square error/variance of the series). Thus, the errors are normalized by the variance of the time series; an *R* ^2^ of 1 indicates perfect forecasts while a value close to 0 or negative indicates poor predictability. Forecasting accuracy was tested for all the fitted models. To establish a baseline for comparison, the predictive *R* ^2^ was also computed when the prediction is the monthly mean value of the transformed time series.

### Robustness of the exogenous forcing by climate

Once the best modeling approach was selected, the robustness of the association between the cases and the exogenous forces *T* and *MEI* was assessed with a non-parametric bootstrap approach based on 10,000 randomizations. The idea of the non-parametric bootstrap is to reconstruct an experimental dataset based on the fitted values of a model plus the residuals sampled with replacement from such a model [24]. To generate the bootstrap samples, the model with the highest predictive *R* ^2^ was used. The bootstrap was initially used to see the frequency (%) of times that the model from which we generated the bootstrap samples was actually selected as the best model, using the Akaike Information criterion [25],[26]. Then, using the sub-sample of models selected as best that also have the highest probabilities in the above bootstrap test, we constructed confidence intervals for the parameters. We further refitted the model without the last 24 points to make forecasts and get the predictive *R* ^2^ confidence intervals.

## Results

**Figure 2:**
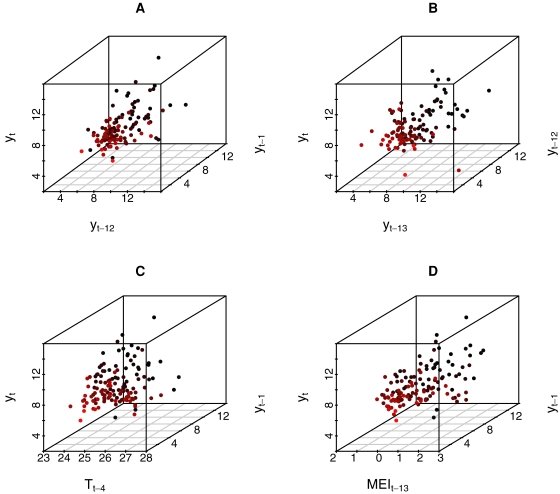
Multidimensional plots for the square root transformed ACL cases (y_t_) as function of lagged components and climatic covariates. (A) Autoregressive (y_t−1_) and Seasonal (y_t−12_) components. (B) Seasonal (y_t−12_) and Autoregressive Seasonal (y_t−13_) components. (C) Autoregressive component (y_t−1_) and Temperature (lag 4, T_t−4_). (D) Autoregressive component (y_t−1_) and MEI (lag 13, MEI_t−13_).

Figure 2 shows the square root transformed cases plotted against their lagged values (1, 12 and 13 months) and the lagged covariates *T* (4 months) and *MEI* (13 months). In all cases, no obvious non-linearity is apparent in the relationship among the three variables. As expected, all models but FNN were most successful for predictions of 1 month ahead. However, for prediction steps larger than one month only NLF, SAR and GAM models with environmental covariates, *MEI* and *T* (4 months lag), did better than predictions based on the average of the time series (Table 1]). The models with the worst performance were FNNs, followed by BSM and the null SAR (i.e., without covariates). For NLF, the best results were found with E = 2 and E = 3, with the latter embedding dimension providing slightly better results for a 12 months ahead prediction.

**Table 1:** Models and predictive *R^2^*

Model	1 month	3 months	6 months	12 months
NLF (E = 2)	0.69	0.62	0.61	0.66
NLF (E = 3)	0.67	0.60	0.59	0.67
NLF (E = 4)	0.66	0.59	0.58	0.66
FNN (2 Layers)	0.55	0.53	0.44	0.44
FNN (3 Layers)	0.62	0.58	0.61	0.60
SAR (null)	0.71	0.64	0.62	0.57
SAR (MEI)	0.73	0.67	0.67	0.66
SAR (MEI+T)	0.77	0.73	0.73	0.72
BSM	0.69	0.59	0.52	0.65
GAM (MEI)	0.66	0.59	0.56	0.57
GAM (MEI+T)	0.73	0.68	0.67	0.68
MEAN	0.64	0.64	0.64	0.64

For model identification, see common abbreviations. Months indicate the number of months predicted ahead. Mean indicates the results that could be obtained by just using the monthly average number of cases.

**Figure 3:**
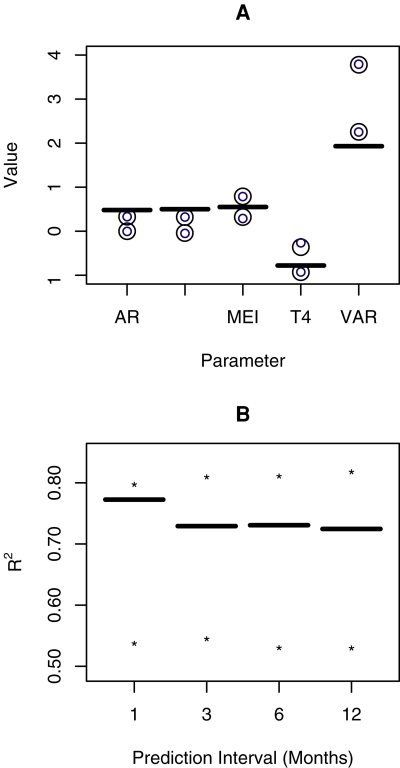
Bootstrap Experiment. (A) 95% Confidence intervals for the parameters of the best model. AR stands for the autoregressive component of the model (*φ_1_*); ARseas for the seasonal autoregressive component of the model (*φ_12_*); VAR for the variance of the residuals (*σ_ε_^2^*); MEI and T4 for the parameter for MEI at lag 13 (*α*) and Temperature at lag 4 (*γ*), respectively. Black signs are 95% confidence intervals using values from the sub-sample when the model is selected as best, and blue including all the bootstrap samples. The structure of the best model can be seen in Protocol S1. (B) Predictive *R* ^2^ and the 95% confidence intervals, indicated by stars, for the bootstrapped best model and prediction interval.

The predictive *R* ^2^ was highest for the SAR model with *T* (4 months lag) and *MEI* (13 months lag) as covariates. Thus, the fitted values and residuals used for the bootstrap were those of the model in the first equation of (1) in Protocol S1. The bootstrap results show that the best model is the one used to generate the data (for 67.40% of the simulated time series, the model was selected as best). The confidence intervals for this model show that the parameters for *T* and *MEI* are statistically significant, a result that holds even if the intervals are constructed using the values for this parameter when the model was not selected as best (Figure 3A). The autoregressive terms, however, are not significant as the confidence intervals include 0. The variance of the residuals obtained from the real data is significantly shorter than the one from the simulations, probably because of the destruction of the autoregressive structure by the re-sampling of residuals [25]. Finally, the results also show (Figure 3B) that the maximum forecasting ability for these models is 80%, and can be as low as 50% probably because of the sensitivity of the models to a lack of a well-defined SAR structure.

## Discussion

The need for forecasts by policy makers goes well beyond the development of EWS for diseases. Due to large-scale, rapid changes, from increased average temperatures to extensive land use changes, major alterations in biogeochemical cycles, water availability, food production, biodiversity and diseases are already occurring and likely to be exacerbated in the future [27,28]. Although the imperative need for predictions that can inform policy has been repeatedly emphasized [11,28], the common practice regarding diseases is to evaluate models by their ability to fit the data [29–35] and only in very few instances have tests been conducted based on data that have not been used to fit the models [6]. Consideration of “out-of-fit” data is critical if we are to evaluate the ability of the models to predict the future.

In this paper, we have presented several methods to study seasonal time series, and used a simple measure, the predictive *R* ^2^, to compare models based on their ability to predict future dynamics and not their goodness of fit of the past. By comparison with modeling results for other infectious diseases on the predictability of NLF methods [36], our results demonstrate a very high predictability for ACL. An important element that might explain this difference is the association of this disease to climate, since models that incorporated climatic covariates performed generally better than those that only considered previous disease levels. Another explanation might be the robustness of the association between the disease and climatic covariates as demonstrated by the bootstrap results. While the parameters for the covariates are statistically significant, the autoregressive parameters are not consistently so, and the variance of the residuals significantly increases.

One of the main lessons from the study of populations is that non-linear dynamics are common in nature but often satisfactorily captured by linear approximations [37,38]. This has been demonstrated by the analysis of time series from a wide variety of animals and diseases. While chaos is present in a small sample of the populations considered, periodicities are common, particularly in infectious diseases, that can be explained by either the effect of exogenous forces, like climate, or endogenous ones, like recruitment of new individuals and the concurrent changes in densities [39–42]. Our results indicate that ACL is another example of a population phenomenon whose dynamics can be satisfactorily described by linear statistical models, provided that appropriate covariates and transformations of the data are used. Thus, though linear models do best, functional forms underlying the influence of covariates are likely to be non-linear as indicated by the transformations used. This result is further supported by the observation that the predictive *R* ^2^ for NLF with E = 3 does not vary with the prediction time step, while this value for the SAR model without covariates decreases abruptly, as expected in systems where the dynamics are non-linear [36–43]. Linear models were also used successfully for other vector-borne diseases, malaria [43] and Ross river virus [4,35], and for cutaneous leishmaniasis in other regions of the new world [45]. For ACL, the usefulness of linear models (after appropriate transformation) might also follow from the fact that humans are only sinks for the pathogen and, therefore, provide no feedback to transmission [46,47]. This conjecture would not necessarily apply to other vector-transmitted diseases in which infected humans provide sources of new infections within the population.

This result also highlights two open questions that need to be addressed when modeling infectious diseases transmitted by vectors: first, the appropriate functional form to introduce climate variables into the dynamics [46,48]; second, the best approach for modeling seasonality [8,49]. Mathematically the relationship between climatic co-variates and the numbers of the disease can be non-linear, described by simple non-linear functions, like those of the functional responses in consumer-resource interactions (e.g., hyperbolic functions) [50] or modeled by linear models with self-excited thresholds [51]. This is especially relevant, since a saturating non-linear functional form can lead to very different scenarios in the dynamics of the disease under altered environmental conditions. In the case of ACL, however, no apparent need for non-linear functions describing the relationship to climate was evident. In general, seasonality has been modeled using fixed structures, i.e., values are assumed to be constant [e.g., 9,49] or approximated by the sum of sine and cosine functions [e.g., 41,52]. The introduction of SAR seasonality in mechanistic models should be further investigated.

A factor that deserves further consideration in developing EWS is the understanding of the role of space. Predictability at more local scales was not addressed here because half of the series was only available at levels below that of the whole country, and because Costa Rica encompasses a small area for which temperature variability is quite homogenous, as seen in the very small variability between temperature grids. However, for larger spatial scales heterogeneities in the landscape for disease transmission would need to be considered [53].

### Conclusions

EWS are a feasible ecological application for neglected tropical diseases, as illustrated for ACL. Available models have good levels of predictability up to one year ahead for the number of cases. Predictability strongly depends on the use of an appropriate structure for the different components of the model, including seasonality and exogenous drivers such as climatic variables. Depending on the model, predictability can range from poor, with approximately 50% accuracy, to high, with 80% accuracy, significantly better than that of seasonal averages (about 65%). Forecasts can be useful in planning services for the populations affected, allowing estimates of approximate number of hospital beds, vaccine shots, drug doses and vector control measures. If EWS need to incorporate the spatial spread of the disease, they should do so dynamically and in relation to different landscapes, such as the geopolitical unit of this study or regions with similar climatic patterns [53]; otherwise, predictions are likely to fail, as illustrated by [12]. While there is no unique early warning system for a given disease, there should be a general approach for the development of EWS. Our work illustrates three key components of such an approach for vector-borne diseases: (i) the evaluation of predictability with “out-of-fit” data and not simply goodness of fit [6,^40^,41]; (ii) the comparison of a suite of possible models in terms of predictability [55,56], and (iii) the robustness of the relationship with covariates in the selected model. Here, robustness is used following [55], to identify covariates that are useful to predict disease numbers even when the skeleton of the model changes. Finally, none of these efforts are possible without the invaluable role of sustained surveillance and monitoring efforts. A historical retrospective reinforces this point: the success of Christophers was possible because of data availability and a deep knowledge of malaria biology, from parasites to mosquitoes and humans, realizing the influence of factors as diverse as weather and wheat prices in rendering the epidemics of malaria predictable [8]. Time series sufficiently long for developing and evaluating forecasting models around the world are countable; their number pales by comparison to the data available for weather forecasting. It is imperative that ongoing efforts are sustained and new ones are initiated whose long-term planning includes EWS as a specific goal.

## Supporting Information

Protocol S1 Linear and non-linear models for time series forecasting.
(0.07 MB DOC)
