# Supplementary material for: Comparing Models for Early Warning Systems of Neglected Tropical Diseases
Source: PLoS Negl Trop Dis. 2007 Oct 22;1(1):e33. doi: 10.1371/journal.pntd.0000033 (PMC2041810; doi:10.1371/journal.pntd.0000033)

**Protocol S1**

**Linear Models**

*Seasonal Autoregressive* (SAR): these models were fitted to the data using Kalman recursions for their state space representation (see Chaves & Pascual, 2006, for technical details). These models incorporate seasonality by considering autoregressive components with the period of the time series under study. For the purpose of comparison, the following three models published in Chaves & Pascual (2006) were considered:

(1)

Where the error terms are assumed to be identical, independent and normally distributed (i.e., i.i.d. normal, ε~N(0,σ^2^) and μ’ = μ + the effects of covariates (γMEI, αT) when considered. These models were fitted using R (R Devolopment Core Team, 2006).

*Basic Structural Model* (BSM): The BSM is one of the simplest linear gaussian state space models. It decomposes an observed time series (*y_t_*) into a local level (*μ_t_*), which basically is a changing mean value or the equivalent to an intercept in least squares regression, a trend (*v_t_*), and seasonal components:

(2)

The equations are solved and smoothed using Kalman recursions. The parameters are the variances of the errors in the four equations, which are assumed to be i.i.d. normal (for details, Brockwell & Davis, 2002; Durbin & Koopman, 2001). The model was also fitted using R (R Devolopment Core Team, 2006).

**Non-Linear Models**

*Generalized Additive Models* (GAM): Additive models are a combination of parametric and non parametric models (Venables & Ripley, 2002; Faraway, 2005). Unlike linear models, were linear parametric forms define the shape of the relationship between responses and predictors, in GAM smooth functions (*f*) that can be far from linear are used for such relationships. In this work, the smooth non-parametric functions were computed using a penalized smoothing spline approach in which the parameters for smoothing where obtained by generalized cross-validation of a function that weights the trade-off between the smoothing and the likelihood of the fitting (for technical details see Faraway, 2005). An intensive process of model selection, based on likelihood tests and the Akaike Information Criterion (e.g., Chaves & Pascual, 2006), led to the selection of the following two best models:

(3)

For the models in (3) the assumptions about the error in (1) hold. These models were fitted using the library mgcv of R (R Devolopment Core Team, 2006)

*Feed-forward Neural Networks* (FNN): Feed-forward neural networks are computer-based models that try to emulate the human brain in performing complex tasks (Venables & Ripley, 2002; Faraway, 2005). In the application of these models to time series analysis, there is no mechanistic interpretation of the layers of neurons. These simply provide a phenomenological and flexible treatment of functional relationships between predictors and responses. In models with one hidden layer of “neurons”, an arbitrary functional form is decomposed into a sum of sigmoids (Ellner et al, 1995):

(4)

Where G is a univariate sigmoid function like the logistic expression e^u^/(1+e^u^). Models with the same predictors as in (1) were fitted and tested using up to 3 neurons. These models do not have explicit assumptions about the errors. Parameters (weights in the FNN’s jargon) are obtained with a search that minimizes residuals over 100000 experimental fittings (e.g., Faraway, 2005). These models were fitted using the library nnet of R (R Devolopment Core Team, 2006). The goodness of fit was highest for models with the same predictors as the ones described in (1).

*Non-linear Forecasting* (NLF): Though originally used to assess the degree of determinism in the dynamics of populations (Sugihara & May, 1990), NLF is ultimately a forecasting tool. It is a technique based on the multidimensional embedding of a time series, where the E-dimensional set of points *x*_t_= (*y*_t_, *y*_t-τ_,…,*y*_t-(E-1) τ_) is constructed and called the phase space, and τ represents the time delay in the observations (e.g. Sugihara & May, 1990; Hegger et al, 1999; Pascual et al., 2000). NLF is implemented by first identifying neighboring points of *x*_t_ in delayed embedding space, and then obtaining a prediction of the response variable at time t+k as simply the average over the future state of the neighbors (Sugihara & May, 1990):

(5)

A key step in using NLF is to choose the appropriate dimension E. One approach is to use several dimensions in order to find the value of E that optimizes predictions (Sugihara & May, 1990). Here we used as a guide for an initial value of E, results from the false nearest neighbors method (Kennel et al., 1992). This method finds the minimum embedding dimension (E), by computing the minimum number of multidimensional points that are erroneously mapped on the neighborhoods of other points when the value of E is diminished from an initial value. For the time delay τ, the most common practices are to assign time delays (τ) of 1 (Sugihara & May, 1990; Grenfell et al, 1994). Here, a criterion based on time delayed average mutual information (AMI) was used (Fraser & Swinney, 1986). This function accounts for linear and non-linear correlations in a time series and is computed as follows:

(6)

Where p_i_ is the probability to find a time series value in the i-th interval of some partition of the data, p_ij_(τ) is the joint probability that an observation falls into the i-th interval and τ times units later, in the j-th interval. The AMI and the false nearest neighbors method were implemented using the library tseriesChaos for R (R Devolopment Core Team, 2006) and the NLF’s were computed using the package predict of TISEAN (Hegger et al., 1999).

**Choosing the Embedding dimensions and lags for the Non-Linear Forecasting**

Figure s1 shows the results of the false nearest neighbors and the average mutual information (AMI) methods. The false nearest neighbors approach suggests embedding dimensions of two, three or four, since larger values do not lead to a considerable decrease in the % of false neighbors, indicating that state-space is successfully unfolded for lower values of E. The AMI method suggests that good lags for the delay τ in the NLF are 1, 5 and 10. Given the limitation of time series length, we chose to set τ=1 and to consider values of two, three and four for the embedding dimension.

**References**

Chaves LF, Pascual M (2006) Climate cycles and forecasts of cutaneous leishmaniasis, a nonstationary vector-borne disease. PLoS Med 3: e295.

Durbin J, Koopman SJ (2001) Time series analysis by state space methods. Oxford: Oxford University Press.

Ellner S, Gallant AR, Theiler J (1995) Detecting nonlinearity and chaos in epidemic data. Epidemic models, their structure and relation to data (D. Mollison Ed), pp, 229-247. Cambridge: Cambridge University Press.

Faraway JJ (2005) Extending the linear model with R: generalized linear, mixed effects and nonparametric regression models. Boca Raton: Chapman & Hall. CRC.

Fraser AM, Swinney HL (1986) Independent coordinates for strange attractors from mutual information. Phys Rev A 33: 1134-1140.

Grenfell BT, Kleczkowski A, Ellner SP, Bolker BM (1994) Measles as a case study in nonlinear forecasting and chaos. Phil Trans Roy Soc London A. 348: 515-530.

Hegger R, Kantz H, Schreiber T (1999) Practical implementation of nonlinear time series methods: the TISEAN package. Chaos 9: 413-435.

Kennel MB, Brown R, Abarbanel HDI (1992) Determining embedding dimension for phase-space reconstruction using a geometrical construction. Phys Rev A 45: 3403-3411.

R Development Core Team. (2006) R: A language and environment for statistical computing. R Foundation for Statistical Computing: Vienna.

Sugihara G, May RM (1990) Nonlinear forecasting as a way of distinguishing chaos from measurement error in time series. Nature. 344: 734-741.

Venables W, Ripley B (2002) Modern applied statistics with S 4^th^ ed. New York: Springer.

**Figure S1** False Nearest Neighbors and Average Mutual Information (AMI) for the square root transformed ACL Cases (*y_t_*)


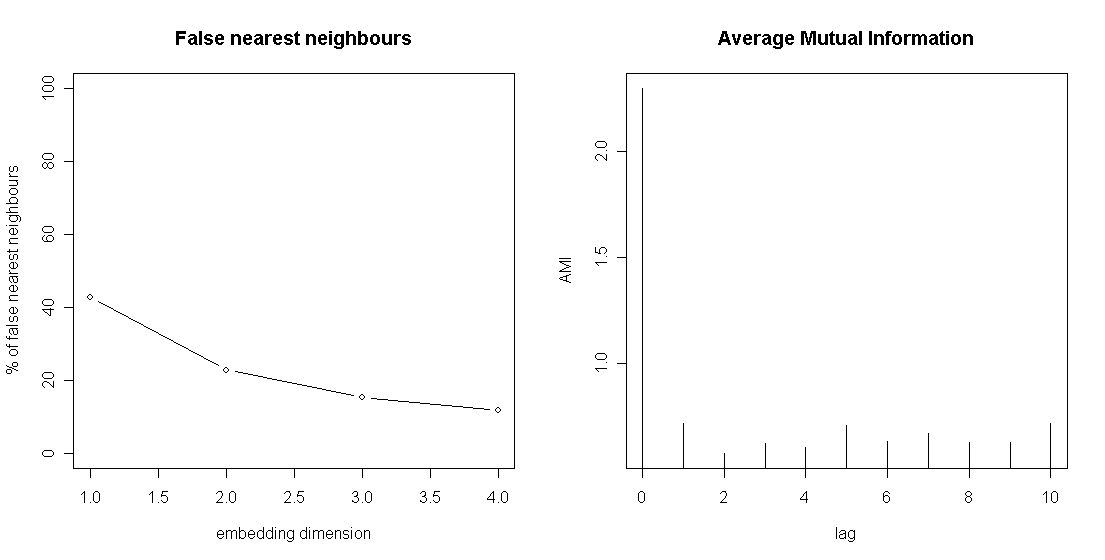

Supplement: Protocol S1 — Linear and non-linear models for time series forecasting. (0.07 MB DOC) [file pntd.0000033.s001.doc]
